# IKZF1 genetic variants rs4132601 and rs11978267 and acute lymphoblastic leukemia risk in Tunisian children: a case-control study

**DOI:** 10.1186/s12881-019-0900-1

**Published:** 2019-10-11

**Authors:** Sana Mahjoub, Vera Chayeb, Hedia Zitouni, Rabeb M. Ghali, Haifa Regaieg, Wassim Y. Almawi, Touhami Mahjoub

**Affiliations:** 1 Laboratory of Human Genome and Multifactorial Diseases (LR12ES07), Faculty of Pharmacy of Monastir, University of Monastir, Monastir, Tunisia; 2 Hematology Department ; Faculty of Medicine Ibn Jazzar, University of Sousse, Sousse, Tunisia; 3 Faculty of Sciences, El-Manar University, Tunis, Tunisia; 4 School of Medicine, Nazarbayev University, Nur-Sultan, Astana, Kazakhstan

**Keywords:** Acute lymphoblastic leukemia, Gene polymorphism, IKZF1, rs4132601, rs111978267

## Abstract

**Background:**

Associations between *IKZF1* gene variants and Acute Lymphoblastic Leukemia (ALL) was recently reported. We examined whether the common *IKZF1* polymorphisms rs4132601 T/G and rs111978267 A/G are associated with ALL among a Tunisian pediatric cohort.

**Methods:**

This case-control study involved 170 patients with ALL and 150 control subjects. SNP genotyping was performed by TaqMan® SNP Genotyping Assay.

**Results:**

The minor allele G of *IKZF1* gene polymorphism rs4132601 T/G was significantly higher in ALL cases than in control subjects (*P* = 0.029), with 1.54-fold increased risk of ALL. The association of rs4132601 with ALL was seen under co-dominant (*P* = 0.009), recessive (*P* = 0.006), and additive (*P* = 0.027) genetic models, of which the co-dominant (*P* = 0.027) and recessive (*P* = 0.027) association remained significant after adjusting for covariates, and False Discovery Rate correction. In contrast, no association was noted for rs111978267 variant. Two-locus (rs4132601-rs11978267) *IKZF1* haplotype analysis demonstrated association of GA (*P* = 0.053), with increased ALL risk [OR (95% CI) = 1.58 (1.00–2.51)], which remained significant after controlling for key covariates [*aP* = 0.046; aOR (95% CI) = 1.61 (1.01–2.57)].

**Conclusion:**

We demonstrated the association of *IKZF1* polymorphism rs4132601 T/G with increased risk of ALL among Tunisian pediatric cohort, with altered phenotypic changes among ALL patients.

## Background

Acute lymphoblastic leukemia (ALL), an aggressive lymphoid cancer, affecting red, white blood cells and platelets. It is reported as the most prevalent hematopoietic malignancy in children worldwide [1, 2]. ALL represent about 25% of cancers that affect people before their twenties; with 2 to 5 years being the peak age. In Tunisia, 25 new children were diagnosed with ALL every year. New treatment strategies have increased the cure rate to 80% [3, 4] and the five-year survival is estimated to 60% nowadays [2]. ALL is characterized by infiltration to monoclonal immature cell medullar and extra medullar areas [5, 6]. ALL is a multifactorial disease that includes the interaction between environmental and genetic factors [7, 8]. Genetic etiology dominates in younger due to lower exposure to environmental factors compared to adults [8, 9].

Recent evidence revealed that acquired somatic mutations could contribute to ALL [10]. These mutations increase the proliferation and survival of progenitor cells [10] and impair further cellular differentiation [10]. Although these alterations contribute to the diagnosis and prognosis [10], they are insufficient to understand the physiopathology ALL processes. The introduction of novel diagnostic tools, such as high-resolution karyotyping, CGH array and genome-wide molecular analysis [6, 11] may reveal additional somatic mutations not captured by standard cytogenetic analysis. The heritable susceptibility to ALL is proven by the finding of recent candidate-gene and genome-wide association studies (GWAS). Some authors suggested that ALL risk is associated with multiple germline variants [12]; others disprove these hypotheses suggesting that the genetic susceptibility to ALL is mediated by a small number of genes with a strong effect rather than the cumulative effect of small genes [13]. In fact, the first GWAS performed by Trevino and colleagues, identified eighteen germline single nucleotide polymorphisms (SNPs) of 307,944 subjects with allele frequencies are higher in pediatric ALL population that in non-ALL controls (*p* < 1 × 10–5) [9]. Four loci particular SNPs located in 7p12.2 (*IKZF1*), 9p12 (*CDKN2A/CDKN2B*), 10q21.2 (*ARID5B*) and 14q11.2 (*CEBPE*) are reported by Sherborne which are associated with ALL risk: RR (1.5–1.6) and more specifically with the B-cell precursor (BCP) ALL [14, 15].

IKAROS one of five members of large Zinc finger proteins (IKAROS, Helios, Aiolos, Eos and Pegasus), and is an important activator in the lymphopoiesis [16]. IKAROS is a potential regulator of lymphocyte commitment and differentiation [17], and immune system development [18]. IKAROS is an important transcription factor expressed in a hematopoietic stem cell that regulates cell proliferation and differentiation during hematopoiesis, particularly in lymphoid cell lineage, and the adaptive immune system [19, 20].

*IKZF1* gene is located at chromosome 7 encodes IKAROS protein interacting with the deoxynucleotidyl transferase inducing its transcription. It especially targets the zone of dead centromeric chromosomes [21]. It has been reported to have a key role in IKAROS protein dysfunction upon mutations in hematological malignancies, especially in lymphoblastic leukemia [17, 22, 23]. Numerous studies documented the association of gene deletion and ALL [24]. Few studies on African and Asian population investigated the association of single polymorphisms of *IKZF1* with infant ALL. Here we explored the association of the *IKZF1* gene variants, rs4132601 located on chromosome 7 (position 50,438,098 between rs6964823 and rs6944602, and strongly associated with ALL), and rs11978267 [25], with ALL risk among Tunisian children.

## Methods

### Subjects

From January 2013 to May 2017, we collected 170 children diagnosed with ALL with a mean age of 7.6 ± 4.5 years old. The immature blood and bone marrow cells were identified after May-Grunwald- Giemsa (MGG) coloring; the ALL classification was made according to the French-American-British (FAB) recommendations (cooperative group) and cytochemical reactions (myeloperoxidase for AML and esterase for ALL), immunophenotyping was done by Beckman cytoflex. Of these patients, 150 were diagnosed with B-ALL and 20 with T-ALL. In addition, 150 children with a mean age of 7.9 ± 5.0 years old served as controls, and were recruited from general pediatric service during a routine check-up and were matched to cases according to self-declared ethnic origin. Blood samples were collected from all participants in ethylenediaminetetraacetic acid containing tubes for further genomic DNA extraction. Written informed consent to participate was obtained from the guardians of all of the patients and controls who participated in this study. The data was performed in accordance with the Declaration of Helsinki which was approved by a ethics committee of Farhat Hached university hospital based in Sousse, Tunisia (12/6/2016),

### SNP genotyping

Total genomic DNA was isolated from peripheral blood leukocytes by the salting-out method. Briefly, blood samples were treated with erythrocyte lysis solutions, followed by protease K digestion. NaCl was added following centrifugation, and the supernatant was treated with absolute ethanol to precipitate DNA. *IKZF1* rs4132601 (170 cases and 150 controls) and rs11978267 (150 cases and 150 controls) genotyping was done using TaqMan® SNP genotyping assay. A standard 10 μl PCR reaction mix consisted of 1X TaqMan® Genotyping Master Mix (Applied Biosystems), 1X SNP Genotyping Assay Mix and 20 ng of genomic DNA. Genotyping of both rs4132601 (3 subjects) and rs11978267 (17 patients) could not be performed because of poor DNA quality.

### Statistical analysis

Statistical analysis was performed using SPSS 23 (IBM, Armonk, NY). Data were expressed as percentages of total (for categorical variables), or mean ± SD (for continuous variables). Student’s t-test was used to determine differences in means, and Pearson χ^2^ test was used to assess inter–group significance. False Discovery Rate (FDR) was performed to control the error type I, using R software version 3.4.3. Power of the study was calculated by Genetic Power Calculator (http://www.bwh.harvard.edu/gpc). Results with a *p*-value < 0.05 were considered as statistically significant. Genotypes were tested for departures from Hardy–Weinberg equilibrium (HWE) in the control population using Haploview 4.2 (www.broadinstitute.org/haploview). All analyses were conducted under additive genetic model. Pairwise linkage disequilibrium (LD) values were calculated with Haploview 4.2, and haplotype reconstruction was performed by the expectation maximization method. Logistic regression analysis was performed in order to determine the odds ratios (OR) and 95% confidence intervals (CI).

## Results

### Study subjects

The characteristics of ALL patients and control subjects are shown in (Table 1). No significant inter-group differences were recorded for age (*P* = 0.687), or gender (*P* = 0.533). All hematological indices were significantly different between ALL patients and control subjects (*P* **<** 0.001).

**Table 1:** Characteristics of study participants

Parameters	Cases (*N* = 170)^c^	Controls (*N* = 150)^c^	*p* ^*a*^
Mean age (years ± S.D.) ^**b**^	7.7 ± 4.5	7.5 ± 4.5	0.820
Age ≤ 10 years ^**c**^	62%	70%	1.000
Gender (M:F) ^**d**^	50%	44.4%	0.797
WBC (× 10^3^ /μl) ^**b**^	69.31 ± 57.7	13.8 ± 1.9	< 0.001
WBC category (×10^3^ /μl)^**c**^			
< 20	60.2%	91.4%	< 0.001
20–100	17.0%	5.6%	
> 100	22.7%	0 (0.0)	
RBC (× 10^6^ /μl)^***b***^	7.8 ± 2.8	10.9 ± 2.1	< 0.001
Platelets count (×10^3^ /μl) ^**b**^	92,8 ± 15	356.4 ± 15.45	< 0.001
Positive family history ^**d**^	9.02%	N.A	N.A
Immunophenotype			
ALL-B ^**d**^	88.3%	N.A	N.A
ALL-T- ^**d**^	11.7%	N.A	N.A
Current status			
In remission	119	N.A	N.A
Relapsed	41	N.A	N.A
Deceased	10	N.A	N.A

Bold types are indicative of significant association *p* > 0.05

*N.A.* Not applicable

^**a**^Student t-test for continuous variables, chi-square analysis for categorical variables; ^**b**^Mean ± SD

^**c**^Number of subjects (Percentage); ^**d**^Percent total

### Association studies

Table 2 summarizes allelic associations and Hardy Weinberg equilibrium between SNPs of the *IKZF1* gene and ALL. Genotypes of rs4132601 (*P* = 0.88), and rs11978267 (*P* = 0.5) *IKZF1* gene variants were in HWE among control population. Minor (G) allele frequencies (MAF) of rs4132601 was higher in patients and exhibited a significant association with ALL [*P* = 0.029; OR (95% CI) = 1.54 (1.04–2.2)], while there was a lack association for rs111978267 A/G with ALL (*P* = 0.086).

**Table 2:** Distribution of IKZF-1 variants alleles in ALL cases and controls

SNP	Position ^a^	Minor Allele	Cases ^b^	Controls^b^	HWE	χ^2^	p ^c^	OR (95% CI)	Power (%)
rs4132601 T/G	50,402,906	G	36%	26%	0.88	< 0.001	0.029	1.54 (1.04–2.28)	78.5
rs11978267 A/G	50,398,606	G	17%	11%	< 0.05	0.283	0.086	1.57(0.93–2.67)	89.5

^a^Location on chromosome based on dbSNP build 125

^b^Minor allele (frequency)

^**c**^Adjusted *p* value, adjusted for age and gender

*HWE* Hardy-Weinberg Equilibrium

*SNP* Single Nucleotide Polymorphism

rs4132601 is significant associated with ALL (*P*<0.05)

According to Table 3*,* crude results analysis had revealed that the rs4132601 T/G variant was positively associated to ALL risk under the co-dominant model for G/G genotype carriers [*P* (=0.006; OR (95%CI) = 4.07 (1.40–11.79)], under the recessive model of inheritance [*P* = 0.0059; OR (95%CI) = 3,80 (1.35–10.67)], and under the additive model of genetic transmission [*P* = 0.027; OR (95%CI) = 1,56 (1.05–2,34)] between ALL patients and control subjects. While significant associations were maintained after adjusting for age and gender covariates, the association under the additive model was lost [*P* 0 0,054) after FDR correction. For rs11978267 A/G variant, there were no significant associations between genotypic distributions and ALL susceptibility under any of the models of inheritance (Table 3).

**Table 3:** Genotypic distributions of the rs4132601 T/G and rs11978267 A/G variants between cases and controls according to different models of genetic transmission

SNP	Model	Genotype	Cases (%)	Controls (%)		OR (95% CI)	*aP* ^*a*^	*P* corr ^b^	aOR (95% CI)^*a*^
rs4132601	Codominant	T/T	43%	51%	–	1.00 (Reference)	**–**	–	1.00 (Reference)
		T/G	43%	45%	0.60	1.15 (0.67–1.96)	0,58	0.696	1.16 (0.68–1.99)
		G/G	14%	4%	**0.006**	**4.07 (1.40–11.79)**	**0,009**	**0.027**	**4.21 (1.42–12.44)**
	Dominant	TT vs. TG + G/G	43.2 vs.56.8%	51.3 vs.48.7%	0.19	1.40 (0.84–2.34)	0.19	0.285	1.41 (0.84–2.36)
	Recessive	TT + TG vs. G/G	86.4% vs.13.6%	95.8)vs. 4.2%	**0.0059**	**3.80 (1.35–10.67)**	**0.006**	**0.027**	**3.91 (1.37–11.19)**
	Over-dominant	T/T + G/G vs. T/G	56.8 vs.43.2%	55.5vs.44.5%	0.79	0.93 (0.56–1.56)	0.83	0.830	0.95 (0.56–1.58)
	Additive	–	–	–	**0.027**	**1.56 (1.05–2.34)**	**0.027**	0.054	**1.58 (1.05–2.37)**
rs11978267	Codominant	A/A	75%	81%	–	1.00 (Reference)	**–**	–	1.00 (Reference)
		A/G	16%	14%	0.56	1.23(0.59–2.55)	0,49	0.588	1.29 (0,61–2.70)
		G/G	9%	5%	0.15	2.20 (0.75–6.43)	0,16	0.30	2.16 (0.73–6.37)
	Dominant	A/A vs. A/G + G/G	74.8 vs.25.2%	81.5% vs.18.5%	0.23	1.47 (0.78–2.75)	0.2	0.30	1.51 (0.80–2.85)
	Recessive	A/A + A/G vs. G/G	90.9% vs.9.1%	95.6 vs.4.4%	0.16	2.13 (0.73–6.18)	0.18	0.30	2.08 (0.71–6.10)
	Over-dominant	A/A-G/G vs. A/G	83.8% vs.16.2%	85.9 vs.14.1%	0.68	1.16 (0.56–2.39)	0.59	0.59	1.22 (0.59–2.55)
	Additive	–	–	–	0.15	1.40 (0.89–2.19)	0.14	0.30	1.41 (0.89–2.22)

^*a*^*aP*, aOR: *p-value* and odds ratio are adjusted by age and gender

^b^ *P* corr.: FDR corrected *p*-value

*SNP* Single Nucleotide Polymorphism

Bold faced values are indicative of significant association (*p* < 0.05)

### Correlation studies

We next examined correlations between *IKZF-1* variants and ALL-associated hematological and biochemical parameters. As shown in Table 4, for the rs4132601, a negative correlation was denoted for ALL-B blood cell number (R = -0.20; *P* = 0.03) and an inverse positive one was demonstrated for ALL-T immunophenotype (R = 0.20, *P* = 0.03). For the rs11978267, nearly significant weak negative correlations were observed for platelet count (R = − 0.096, *P* = 0.14) and LDH parameters (R = − 0.286, *P* = 0.18) (Table 4).

**Table 4:** Pearson correlations of *IKZF1* studied variants

Factor	rs4132601		rs11978267	
	*P*	R	*P*	R
Gender	0.26	0.10	0.52	−0.06
Immunophenotype				
ALL-B	**0.03**	**0.20**	0.20	0.12
ALL-T	**0.03**	**−0.20**	0.20	−0.12
WBC	0.40	0.054	0.20	−0.04
Platelets count	0.53	−0.04	**0.14**	**−0.09**
LDH	0.25	−0.21	**0.18**	**−0.28**
Current status	0.89	−0.01	0.30	−0.11

*P*: *p*-value

R: Pearson correlation coefficient

Bold –faced values are indicative of a significant association

*WBC* White Blood Cells

*LDH* Lactate dehydrogenase

### Haplotype analysis

Two-locus [rs4132601 and rs11978267] *IKZF-1* haplotype analyses denoted that the majority of the haplotype diversity was captured by 4 haplotypes in controls (96.1%) and cases (100%), namely TA, GA, GG, TG. Taking the common TA haplotype as reference (OR = 1.00), univariate and multivariate analyses demonstrated an association of GA [*P* = 0.053; OR (95%CI) = 1.58 (1.00–2.51)] haplotypes with ALL (Table 5). This remained significant after controlling for age and gender [*aP* = 0.046; aOR (95%CI) = 1.61 (1.01–2.57)].

**Table 5:** IKZF-1 haplotypes distributions in cases and controls

Haplotype^*a*^	Cases	Controls	*P*	OR ^*b*^ (95% CI)	*aP*	aOR ^*b*^ (95% CI)
T A	0.571 ^c^	0.690	–	1.00 (Reference)	–	1.00 (Reference)
G A	0.256	0.193	**0.053**	**1.58 (1.00–2.51)**	**0.046**	**1.61 (1.01–2.57)**
G G	0.1	0.068	**0.095**	**1.77 (0.91–3.46)**	**0.089**	**1.8 (0.92–3.55)**
T G	0.071	0.047	0.33	1.43 (0.69–2.96)	0.37	1.4 (0.68–2.89)

^a^ Haplotype containing rs4132601 T/G and rs11978267 A/G

^b^ *aP*; *aOR* adjusted odds ratio; covariates that were controlled for were age and gender

^c^ Haplotype frequency determined by the maximum likelihood method

Bold-faced values are indicating a significant association

## Discussion

Genetic studies contributed to the understanding of the physiopathological mechanisms of malignant diseases such as leukemia. In fact, the genetic alterations are a major etiology of children leukemia which helps to explain the potential risk for ALL. Xu and colleagues hypothesized that the genetic susceptibility to ALL is mediated by a small number of genes with strong effect rather than the cumulative effect of small genes [13].

Genome association studies have identified the *IKZF-1* gene polymorphism frequency through the deletion and single mutations in the children with ALL as compared to the controls [24–26].

We examined the association between rs4132601 and ALL risk in 170 Tunisian pediatric patients. Our results (Table 3) confirm those of Papaemmanuil and colleagues, who reported the association of the *IKZF1* gene polymorphism rs4132601, localized in 3′ region of the IKAROS family zinc finger 1 (*IKZF1*) gene with a pediatric ALL risk [25]. It is in LD with rs6964823 and rs6944602 that form a block between 3′ UTR and intron 7 of *IKZF1* [25]. However, previous studies that have examined the association between *IKZF1* rs4132601 polymorphism and acute lymphoblastic leukemia (ALL) risk reached controversial results. Li et al. analyzed 15 case-control studies with 8333 cases and 36,036 controls in a meta-analysis [26]. The results suggested that rs4132601 was associated with an increased ALL risk. Significant associations were found among Caucasians and Hispanics [27, 28], but not among Asians [29]. On the other hand, when data are stratified by disease type, rs4132601 increased significantly risks in B-cell ALL and B hyperdiploid ALL, but not among T-cell ALL and AML subgroups [26–30]. Our results showed that the G allele of rs4132601 is more frequent in patients than the control, which increased the ALL risk in Tunisian children. However, Dai and colleagues suggested in their meta-analysis that Europeans but not Asian G allele carriers children had a markedly increased risk of ALL [30, 31].

Several studies have been conducted to examine the possible interaction between *IKZF1* G allele of rs4132601 and environmental ALL risk factors. Rudant and colleagues reported a negative correlation of *IKZF1* allele risk with maternal use of home insecticides during pregnancy and positive interactions with early immune modulation, breastfeeding, and history of repeated early common infections, but there was no risk with paternal preconception smoking [32]. Rudant and Linabery noted that some selected demographic factors such as birth weight and maternal age don’t change the risk of some SNPs of *ARID5B* and *IKZF1* [32, 33]. Also, Linabery and Evans in their independent studies showed an evident interaction between rs4132601 risk and paternal preconception smoking or maternal use of foli *IKZF1*c acid [33, 34].

The rs4132601 is a substitution polymorphism (T > G) located in 3′UTR to the IKAROS Family zinc finger 1 (*IKZF1)* gene (located on 7p12.2). IKAROS proteins are major regulators of lymphocyte maturation and CD4, CD8 T-cell lineage differentiation [35]. The animal model revealed that mice without IKAROS protein develop aggressive lymphoblastic leukemia [36, 37]. However, the role of the rs4132601 in the leukemogenesis is unclear; Górniak and colleagues hypothesized that the G allele decreases the stability and level of the mRNA expression; which delays progression through B-cell maturation and increases the risk of ALL [25, 38]. Lautner-Csorba and colleagues reported that the rs4132601 strongly increases the probability (0.97) of ALL and showed that the lower expression associated with the rs4132601 might contribute to the increased vulnerability to the disease [39]. Our results had disapproved of this hypothesis. The Pearson test did not demonstrate any correlation between rs4132601 and current state (death, relapse) (Table 5).

The rs11197867 *IKZF1* gene variant, the second investigated polymorphism, is an intronic polymorphism located at the position 50,433,798 of chromosome 7. Our data showed the lack of association of rs11197867with ALL, irrespective of the genetic model used. To our knowledge, few studies have replicated this polymorphism. Dai and colleagues in their meta-analysis of 20 studies with 4960 patients and 28,034 controls reported that the rs11978267 polymorphism in the *IKZF1* gene increased significantly AL risk [31], especially among Europeans [28, 33, 40] but not among African and mixed populations [13]. When stratified by types of ALL, a significant correlation was found in the BCP-ALL sub-group in the allelic and ALL genetic models. Moreover, ethnicity appears to change ALL risk. In fact, Europeans with a G allele had a markedly increased risk of ALL, which was not observed among Asians [30].

Mixed Lineage Leukemia (MLL) gene rearrangements assigns a poor prognostic [41] in both ALL and AML infant cases. In fact, MLL status markedly influences the five-year survival rates for infant ALL. It’s reported that 80% of ALL infants with negative MLL rearrangement live up to 5 years or more. The risk death increase with ALL/MLL+. However, this influence is less pronounced in infant AML. Further, ALL/MLL+ infants are usually diagnosed as early as the age of 6 months while those with ALL/MLL− are often depicted in older infants [41]. Burkhardt asserted that the combined cytogenetic MLL with a homozygote variant *IKZF1* allele (rs1197867) increases infant leukemia risk. The risk is similarly increased among young patients with AML/MLL+ and AML/MLL- but only among young patients with ALL/MLL+ [42].

Papaemmanuil and colleagues showed a functional effect for rs4132601, which is in complete linkage disequilibrium with rs11978267, since each additional copy of the variant allele resulted in greater attenuation of *IKZF1* mRNA expression, and consequently induced leukemia [25].

The *IKZF1* gene is polymorphic. Churchman and al. identified 28 germline *IKZF1* variants in children with ALL, mostly in B-ALL [43]. The Iranian (Persian) study including four polymorphisms of the *IKZF1* gene rs4132601 T > G, rs11980379 T > C, and rs10272724 T > C, rs11978267 A > G showed that the first three are associated with risk ALL under the dominant model and that rs11978267 A / G is not associated [44]. Moreover, Oris et al. reported that the four polymorphisms studied rs696823, rs11978267, rs4132601 and rs6944602 are risk factors for ALL [28]. Yemeni study has noted the association between rs10235796 and rs6964969 variants and ALL, with a borderline association denoted for *IKZF1* rs4132601 T/G variant and a lack of association between rs11978267, rs7789635 and ALL [45].

## Conclusion

Our study confirms that the *IKZF1* rs4132601 T/G variant is the risk factor of ALL in the Tunisian pediatric cohort but not the rs11978267 A/G variant. Our results contrivers some other, this might be partly attributed to small sample sizes and ethnic differences across studies. In the other hand, the genetic alterations of *IKZF1* could contribute to the prognosis so that the rs4132601 is associated with ALL immunophenotypes. Our study has several strengths, notably it is one of the few North-African studies replicating such polymorphisms with a considerable statistical power. Further studies with larger sample sizes and more polymorphisms are needed to confirm the role of the IKZF1 gene in the pathophysiology of ALL.

## Data Availability

The datasets used and/or analysed during the current study available from the corresponding author on reasonable request. You could contact Pr Touhami Mahjoub: email: touhamimahjoub@gmail.com

## Abbreviations

ALL: Acute Lymphoblastic Leukemia
AML: Acute Myeloid leukemia
ARID5B: AT-Rich Interaction Domain 5B
B-ALL: B-cell Acute Lymphoblastic Leukaemia
CD: Cluster of Differenciation
CDKN2A: Cyclin-Dependent Kinase Inhibitor 2A
CDKN2B: Cyclin-Dependent Kinase Inhibitor 2B
CEBPE: CCAAT/Enhancer Binding Protein
FDR: False Discovery Rate
GWAS: Genome-Wide Association Studies
HWE: Hardy–Weinberg equilibrium
IKZF1: IKAROS Family Zinc Finger 1
LD: Linkage disequilibrium
MLL: Mixed Lineage Leukemia
WHO: World Health Organization
